# *In vitro* Modulation of the LPS-Induced Proinflammatory Profile of Hepatocytes and Macrophages- Approaches for Intervention in Obesity?

**DOI:** 10.3389/fcell.2016.00061

**Published:** 2016-06-22

**Authors:** Ramiar K. Kheder, James Hobkirk, Cordula M. Stover

**Affiliations:** ^1^Department of Infection, Immunity, and Inflammation, University of LeicesterLeicester, UK; ^2^College of Nursing, University of RaparinRanya, Iraq; ^3^Department of Sport, Health, and Exercise Science, University of HullHull, UK

**Keywords:** Vitamin D_3_, docosahexaenoic acid, HepG2, J774, lipopolysaccharide

## Abstract

Low grade endotoxemia is a feature of obesity which is linked to development of steatohepatitis in non-alcoholic fatty liver disease. In this study, macrophages (J774) and hepatocytes (HepG2) were stimulated with lipopolysaccharide (LPS) from *E. coli* 0111: B4 and analyzed for modulation of this response when preconditioned or stimulated subsequent to LPS, with different doses of Vitamin D_3_ or docosahexaenoic acid (DHA) over a time period of 1 and 5 days. Pro-inflammatory TNFα and pro-fibrotic TGFβ released into the supernatants were measured by ELISA; qPCR was performed for Srebp-1c and PPARα mRNA (genes for products involved in fatty acid synthesis and catabolism, respectively). Vitamin D_3_ and DHA exerted a consistent, dose dependent anti-inflammatory effect, and increased PPARα relative to Srebp-1c in both cell types. By contrast, addition of free fatty acids (FFA, oleic acid/palmitic acid 2:1) caused aggravation of LPS-induced inflammatory reaction and an increase of Srebp-1c relative to PPARα. Our results argue in favor of dietary supplementation of Vitamin D_3_ or DHA (and avoidance of monounsaturated/saturated fatty acids) to alleviate development of fatty liver disease.

## Introduction

Low grade endotoxemia has been detected in circumstances as diverse as a bolus of high fat diet (Erridge et al., 2007), hemodialysis (Terawaki et al., 2010), and surgery (Fujita et al., 2003). Drained via the portal vein, abdominally derived endotoxin, a pathogen associated molecular pattern, PAMP, directly stimulates the reticuloendothelial system of the liver but also hepatocytes. Obesity, from which insulin resistance develops, is linked to increased translocation of endotoxins from the gut (Brun et al., 2007). Steatosis is the accumulation of fat in hepatocytes which can lead to an overall increase in liver size, so-called hepatomegaly. Accumulation of lipids may lead to inflammation; this is called non-alcoholic steatohepatitis, to differentiate the disease from alcohol-induced liver injury, either of which can progress to cirrhosis. It is estimated that there is evidence of non-alcoholic steatohepatitis in up to a third of populations in the developed world (Preiss and Sattar, 2008). Because of increasing prevalence of obesity in these populations, fatty liver disease has recently become a pediatric diagnosis (Giorgio et al., 2013). Bacterial overgrowth, increased gut permeability and intestinal dysmotility are characteristics of patients with non-alcoholic fatty liver disease (NAFLD) and non-alcoholic steatohepatitis (NASH). Endotoxin levels were elevated in patients diagnosed with NAFLD compared to control subjects (Harte et al., 2010). One approach of modulating hepatic disease activity is to target the microbiome (Zhu et al., 2014), but another might be to modulate the effects of bacterial lipopolysaccharide (LPS) on hepatic cells, which express its receptor, TLR4 (Sharifnia et al., 2015). Of note, circulating free fatty acids may also function as endogenous ligands for TLR binding as so-called danger associated molecular patterns, DAMPs.

Vitamin D_3_ and docosahexaenoic acid (an omega 3 fatty acid) offer promise for the adjuvant treatment of NAFLD (Nobili et al., 2011; Eliades and Spyrou, 2015). In fact, Vitamin D_3_ deficiency has been found in patients with NAFLD, and omega 3 fatty acid deficiency is associated with the development of insulin resistance, fatty liver disease, and dyslipidemia (Targher et al., 2013).

Therefore, the aim of this study was to expose macrophages and hepatocytes to LPS and to gauge preventative or curative effects of Vitamin D_3_ and docosahexaenoic acid (DHA) *in vitro* while quantifying a detrimental effect of FFA, a mixture of oleic acid and palmitic acid. Reactions were assessed by measuring TNFα and TGFβ in supernatants of cells for which mRNA expressions for Srebp-1c and PPARα were determined in parallel.

## Materials and methods

### Materials

LPS *E. coli* 0111:B4 was from Invivogen (Toulouse, France), docosahexaenoic acid, oleic acid, and palmitic acid were purchased from Sigma-Aldrich Company Ltd. (Dorset, UK), and Vitamin D_3_ was from DSM Nutritional products (Basel, Switzerland).

### Cell culture

J774, a mouse macrophage cell line, and HepG2, a human hepatocellular carcinoma cell line, were grown to semi confluence at 37°C in a humidified atmosphere (5% CO_2_) in RPMI 1640 medium or DMEM, respectively, supplemented with 10% (v/v) fetal calf serum, 100 μg/ml streptomycin, 100 IU/ml penicillin. They were passaged using trypsin EDTA. Cells were counted and adjusted to 60,000/ml for J774, 40,000/ml for HepG2 in 25 cm^2^ flasks (5 ml) and treated when 70% confluent.

### Experimental design

The doses of Vitamin D_3_ (0.4, 2, 4 μg/ml) were chosen based on a pilot experiment that investigated induction of insulin receptor mRNA in target cells, a described, beneficial effect of Vitamin D_3_ aimed at increasing insulin sensitivity (Maestro et al., 2000; Supplementary Figure 1). DHA was efficient at 50 μM (16 μg/ml) to dampen the proinflammatory response of J774 induced by LPS (100 ng/ml) after 5 days of stimulation (Oliver et al., 2012). Doses of 8, 16, 32 μg/ml DHA were chosen for this work. The composition of FFA was oleic acid/palmitic acid (2:1) and was added at 15 and 30 mM (Yao et al., 2011).

Two basic stimulation models were performed on HepG2 and J774, namely, independently, preconditioning and subsequent stimulation with LPS as well as initial stimulation with LPS and *post-hoc* stimulation: In the preconditioning model, J774 and HepG2 were exposed to different doses of Vitamin D_3_ (0.4, 2, 4 μg/ml), docosahexaenoic acid, DHA (8, 16, 32 μg/ml) or FFA, free fatty acids (oleic acid/palmitic acid 2:1, 15, and 30 mM) prior to stimulation with LPS *E. coli* 0111:B4 (100 ng/ml) for 24 h. In the *post-hoc* stimulation model, J774 and HepG2 were exposed to different doses of Vitamin D_3_ (0.4, 2, 4 μg/ml), docosahexaenoic acid, DHA (8, 16, 32 μg/ml) or FFA, free fatty acids (oleic acid/palmitic acid 2:1, 15, and 30 mM) after stimulation with LPS *E. coli* 0111:B4 (100 ng/ml) for 4 h. While Vitamin D3 and DHA are protective agents, FFA simulates an obesity relevant aggravating agent. Unstimulated controls were run in parallel.

### CDNA synthesis and qPCR

RNA was prepared using RNeasy Mini Kit (Qiagen, Manchester UK), genomic DNA was digested and cDNA was synthesized using RevertAid H Minus First Strand cDNA Synthesis Kit (Thermo Fisher Scientific, Loughborough, UK). Primer sequences are available in Supplementary Table 1. Their efficiency was tested and sensitivity of amplification verified by linearity of product yield vs. dilutions of cDNA. Real time PCR was used to quantify the relative changes in gene expression by 2^−ΔΔCt^ method using SYBR Green formulation and Corbett, Rotor gene 6000 real time rotary analyser. GAPDH mRNA expression was found to be stable. Any variation was corrected by relating expression of gene of interest to GAPDH mRNA expression of the respective controls as part of the 2^−ΔΔCt^ method (Livak and Schmittgen, 2001).

### ELISA

ELISAs for mouse and human Transforming growth factor β, TGFβ, were from R&D Systems (Abingdon, UK), ELISAs for mouse and human Tumor necrosis factor α, TNFα, were from PeproTech EC Ltd (London, UK). Supernatants were used neat and assayed in triplicate. 3,3′,5,5′-Tetramethylbenzidine (TMB) Liquid Substrate System was used.

### Statistics

For ELISA measurements, standard curves were constructed according to the manufacturers' instructions and levels in supernatants extrapolated using Graph Prism Pad. For qPCR analyses, the data were presented as the fold change in gene expression normalized to GAPDH and relative to unstimulated control. Data from triplicate determinations were expressed as means from averages ±SD. Unpaired one-way ANOVA with Tukey's multiple comparisons testing was performed and adjusted *p*-values expressed.

## Results

J774 and HepG2 were used as model cells to investigate in parallel the modulation of their LPS induced response by the presence of Vitamin D_3_ and DHA using markers of relevance in the inflammatory, profibrotic, and lipogenic response. Free Fatty Acids, FFA (oleic acid/palmitic acid 2:1), were used as a stimulus to aggravate LPS-induced reactivity. A special focus was to capture adaptation of the cells to long term corrective exposure with Vitamin D_3_ or DHA before and after LPS stimulation with a low endotoxin dose or the detrimental accumulating effect of FFA when cells were exposed before or after the LPS stimulus.

The dose of LPS *E. coli* 0111:B4 to model responses toward low grade endotoxemia was compared in a pilot experiment to a 10-fold higher dose. The dose of 1000 ng/ml was found to negatively impact TLR and VDR mRNA expression in J774, which behaved as acute sensors of the presence of this PAMP in comparison with HepG2 (Supplementary Figure 2). The dose of 100 ng/ml LPS has been used by others to study the anti-inflammatory effect of DHA on macrophages (Mullen et al., 2010). To determine that the dose of FFA was not toxic, a pilot experiment investigated the effect of FFA on a morphological feature of HepG2 cells, namely lipid accumulation. FFA was found to enhance Oil Red O positive cell inclusions, which could be reduced in the presence of Vitamin D_3_, whilst retaining cell intactness (Supplementary Figure 3). DHA (50 μM) was shown to have a similar effect on palmitate-induced lipids in HepG2 (Luo et al., 2012).

### Benefical effect of DHA and vitamin D_3_ on LPS-induced TNFα and TGFβ production by macrophages and hepatocytes

**Figure 1:**
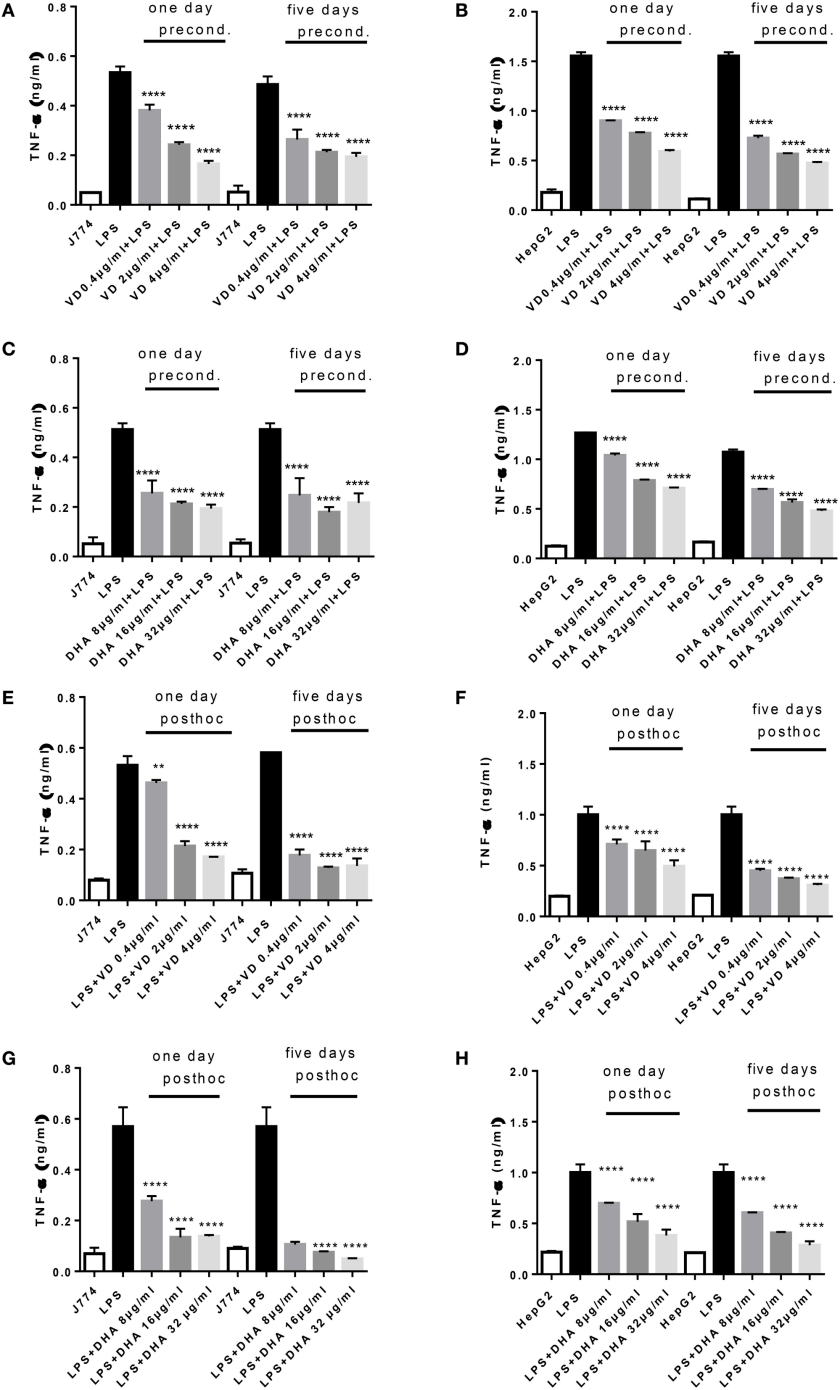
**(A–H)** TNFα response of J774 **(A,C,E,G)** and HepG2 **(B,D,F,H)** toward LPS and its modulation by preconditioning with Vitamin D_3_ **(A,B)** or DHA **(C,D)** or *post-hoc* (subsequent to LPS) stimulation with Vitamin D_3_ **(E,F)** or DHA **(G,H)**. Asterisks indicate significant differences to the LPS induced response. Results are presented as averages ±SD from triplicate determinations. ^**^p < 0.01, ^****^p < 0.0001 (adjusted *p*-values).

A robust induction of TNFα production by 24 h stimulation with LPS was observed in J774 and HepG2 (Figures 1A–D). There was a significant and dose dependent reduction when parallel cultures of J774 or HepG2 were preconditioned with varying concentrations of Vitamin D_3_ (0.4, 2, 4 μg/ml) or DHA (8, 16, 32 μg/ml) for 1 or 5 days. Five days' preconditioning with Vitamin D_3_ (0.4, 2, 4 μg/ml) or DHA (8, 16, 32 μg/ml) before stimulation with LPS was not more effective in reducing the inflammatory gene response than preconditioning for 1 day only. The TNFα response in J774 and HepG2 elicited by an acute 4 h-stimulation with LPS was significantly reduced at 1 and 5 days of subsequent incubation with Vitamin D_3_ or DHA at three different doses each (Figures 1E–H). Macrophages at 5 days' stimulation with Vitamin D_3_ or DHA seemed particularly depressed in their TNFα production (Figures 1E,G).

**Figure 2:**
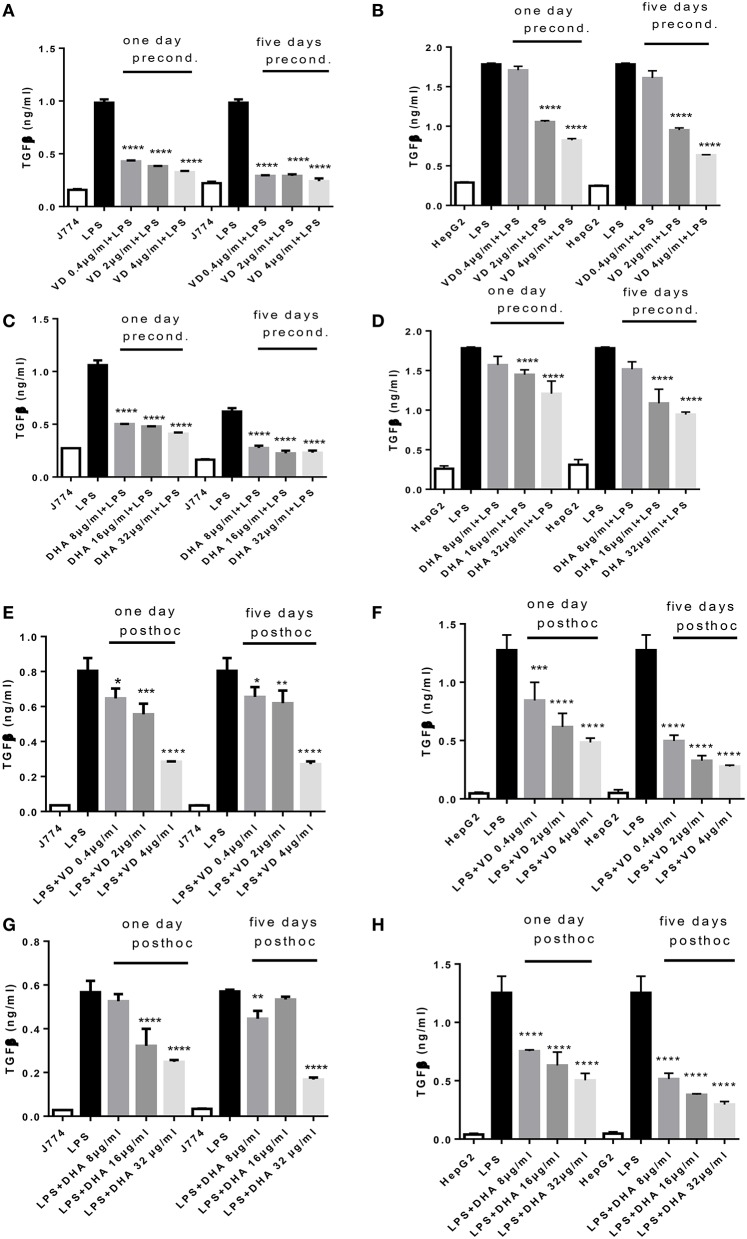
**(A–H)** TGFβ response of J774 **(A,C,E,G)** and HepG2 **(B,D,F,H)** toward LPS and its modulation by preconditioning with Vitamin D_3_ **(A,B)** or DHA **(C,D)** or *post-hoc* (subsequent to LPS) stimulation with Vitamin D_3_ **(E,F)** or DHA **(G,H)**. Asterisks indicate significant differences to the LPS induced response. Results are presented as averages ±SD from triplicate determinations. ^*^p < 0.05, ^**^p < 0.01, ^***^p < 0.005, ^****^p < 0.0001 (adjusted *p*-values).

Analysis of the same supernatants for TGFβ showed that Vitamin D_3_ and DHA significantly reduced TGFβ production of J774 and HepG2 when preconditioned with these modulators prior to LPS stimulation (Figures 2A–D). LPS led to a robust production of TGFβ in macrophages and hepatocytes. It is known that LPS can trigger TGFβ production in monocytes (Toossi et al., 1996). Macrophages at 5 days' stimulation with Vitamin D_3_ or DHA seemed particularly depressed in their TGFβ production (Figures 2A,C). Addition for 1 day and 5 days of higher doses of Vitamin D_3_ (2, 4 μg/ml) or DHA (16, 32 μg/ml) prior to the LPS stimulation reduced the production of TGFβ by HepG2 (Figures 2B,D). LPS-induced TGFβ production by J774 and HepG2 cells was reduced in a dose dependent manner by the subsequent addition of Vitamin D_3_ or DHA for 1 and 5 days (Figures 2E–H). HepG2 seemed more sensitive to the action of Vitamin D_3_ or DHA compared to J774.

**Figure 3:**
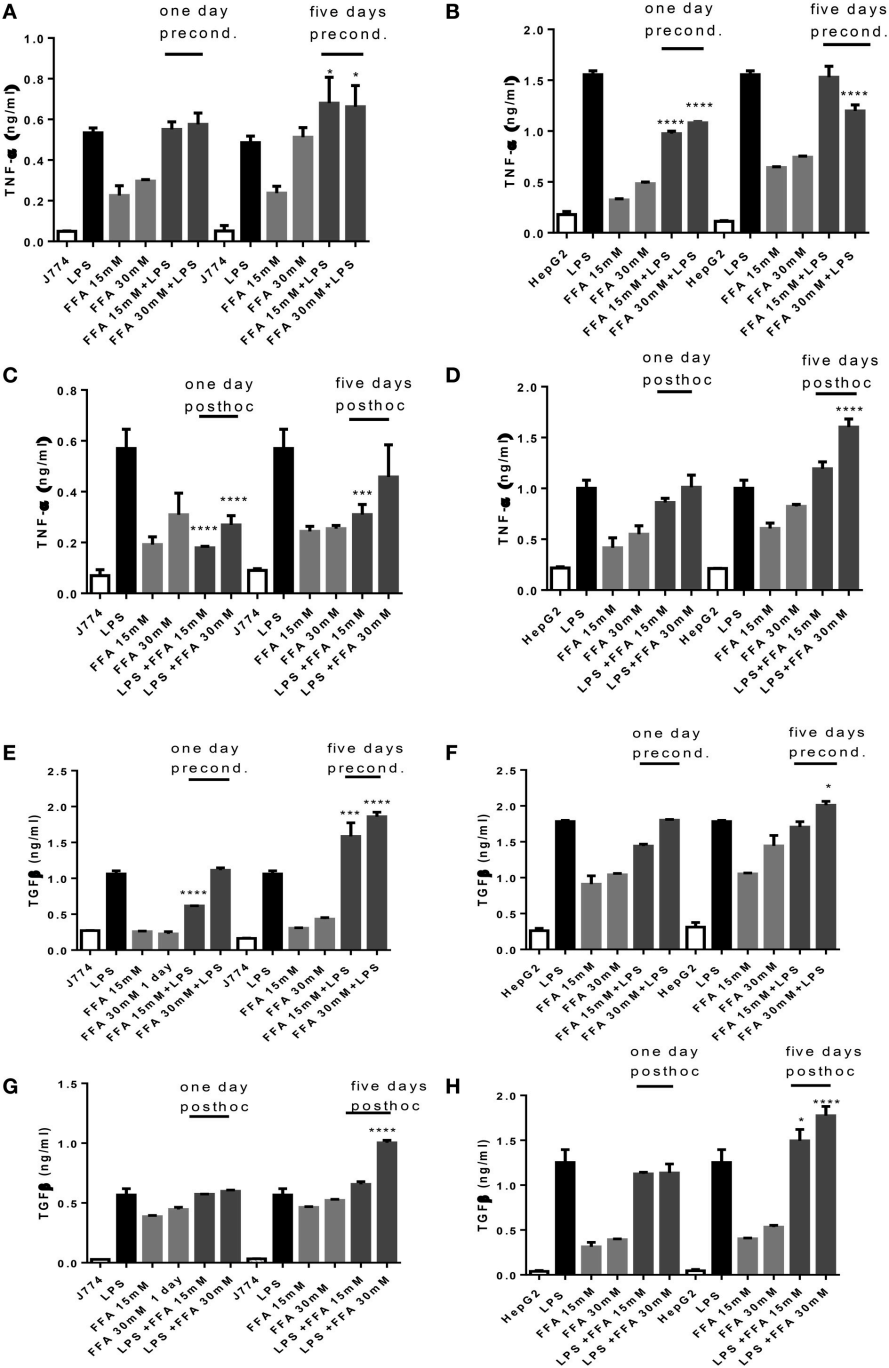
**(A–H)** TNFα and TGFβ response of J774 **(A,C,E,G)** and HepG2 **(B,D,F,H)** toward LPS and its modulation by preconditioning with FFA **(A,B,E,F)** or *post-hoc* (subsequent to LPS) stimulation with FFA **(C,D,G,H)**. Asterisks indicate significant differences to the LPS induced response. Results are presented as averages ±SD from triplicate determinations. ^*^p < 0.05, ^***^p < 0.005, ^****^p < 0.0001 (adjusted *p*-values).

Incubation with FFA elicited a dose dependent TNFα release from J774 and HepG2 compared to basal levels (Figures 3A–D). When preconditioned with FFA, macrophages showed a TNFα response to subsequent LPS stimulation which was of the same magnitude as LPS alone (Figure 3A). Stimulation with FFA subsequent to LPS, however, led to an impaired TNFα response (Figure 3C). In HepG2, stimulation with 30 mM FFA for 5 days prior to LPS exposure, produced TNFα levels which were reduced compared to those generated by LPS alone (Figure 3B), while subsequent exposure with FFA after LPS stimulation further increased TNFα secreted by HepG2 compared to LPS stimulated cells (Figure 3D). Regarding TGFβ, the levels produced by J774 were highest after 5 days of either preconditioning of cells with 30 mM FFA before LPS stimulation, or *post-hoc* treatment of cells with 30 mM FFA after LPS stimulation (Figures 3E,G). The same observation was made for HepG2 cells: stimulation with 30 mM FFA for 5 days prior to LPS exposure, produced TGFβ levels which exceeded those generated by LPS alone (Figure 3F), as did *post-hoc* exposure with 30 mM FFA after LPS (Figure 3H).

### Effect of DHA and vitamin D_3_ on LPS-induced mRNA expression of Srepb-1c and PPARα by macrophages and hepatocytes

**Figure 4:**
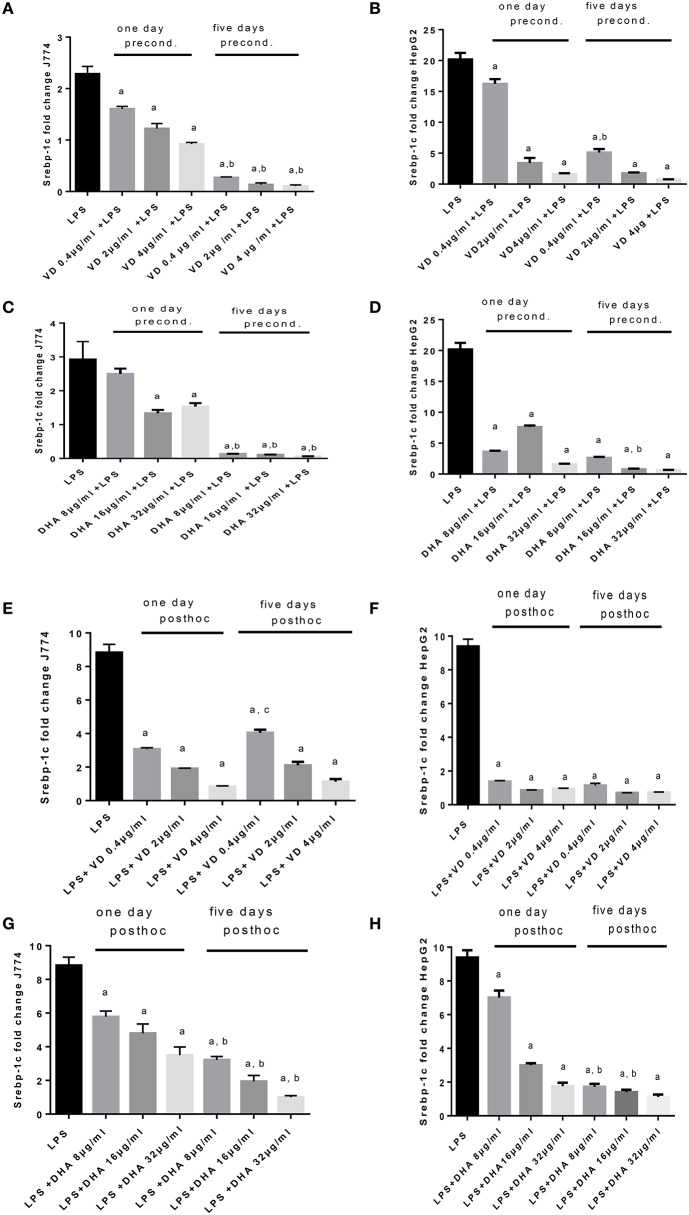
**(A–H)** qPCR analysis of J774 **(A,C,E,G)** and HepG2 **(B,D,F,H)** for Srebp-1c mRNA expression induced by LPS and modulated by preconditioning with Vitamin D_3_ **(A,B)** or DHA **(C,D)** or *post-hoc* (subsequent to LPS) stimulation with Vitamin D_3_ **(E,F)** or DHA **(G,H)**. Triplicate data were averaged, normalized to GAPDH mRNA abundance and expressed in relation to baseline Srebp-1c mRNA expression. ^a^*p* < 0.0001 compared to LPS, ^b^*p* < 0.0001 compared to 1 day precond, ^c^*p* < 0.002 compared to 1 day *post-hoc* stimulation (adjusted *p*-values).

LPS exposure of J774 and HepG2 stimulated Srebp-1c mRNA expression (Figure 4) as expected (Costales et al., 2013). Preconditioning of J774 for 1 day with Vitamin D_3_ led to a dose dependent reduction of Srepb-1c mRNA, which was further reduced after 5 days' preconditioning. This means that 5 days of incubation with a minimum dose of Vitamin D_3_ (0.4 μg/ml) prevented the induction of Srepb-1c observed after addition of 100 ng/ml LPS for 24 h (Figure 4A). The same pattern was observed for HepG2 cells, although higher doses of Vitamin D_3_ (2, 4 μg/ml) were efficient after just 1 day of preconditioning (Figure 4B). Preconditioning of J774 and HepG2 with DHA for 5 days efficiently downregulated Srepb-1c mRNA with a minimum dose studied of 8 μg/ml (Figures 4C,D). One day prestimulation with 8 μg/ml DHA significantly reduced Srepb-1c mRNA in HepG2, while higher doses were necessary to achieve this effect in J774 (Figures 4C,D). Exposure of LPS stimulated J774 with Vitamin D_3_ or DHA yielded a dose dependent reduction in Srepb-1c mRNA for both durations of incubation, 1 and 5 days (Figures 4E,G). Higher doses of Vitamin D_3_ (2, 4 μg/ml) were needed to maintain the reduction in Srepb-1c mRNA observed at 1 day *post-hoc* stimulation compared to 5 days (Figure 4E). DHA, by contrast, significantly downregulated Srepb-1c mRNA at 5 days' incubation after LPS stimulation compared to 1 day incubation (Figure 4G). In the presence of Vitamin D_3_, LPS-induced Srepb-1c mRNA expression was effectively downregulated at 1 and 5 days' stimulation in HepG2 (Figure 4F), whereas higher doses of DHA had this effect at 5 days (Figure 4H).

**Figure 5:**
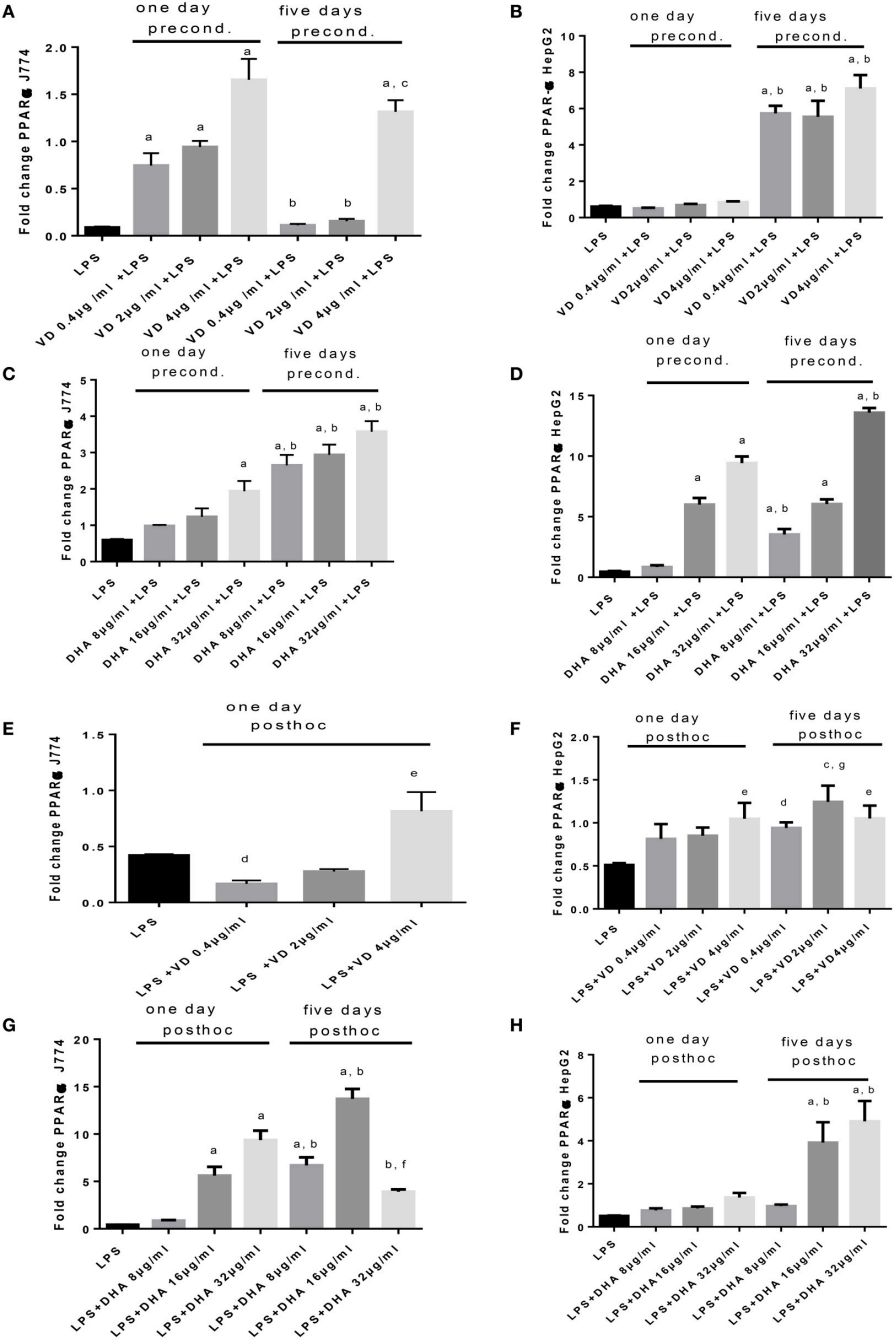
**(A–H)** qPCR analysis of J774 **(A,C,E,G)** and HepG2 **(B,D,F,H)** for PPARα mRNA expression induced by LPS and modulated by preconditioning with Vitamin D **(A,B)** or DHA **(C,D)** or *post-hoc* (subsequent to LPS) stimulation with Vitamin D_3_**(E,F)** or DHA **(G,H)**. Triplicate data were averaged, normalized to GAPDH mRNA abundance and expressed in relation to baseline PPARα mRNA expression. ^a^*p* < 0.0001 compared to LPS, ^b^*p* < 0.0001 compared to day 1, ^c^*p* < 0.05 compared to day 1, ^d^*p* < 0.05 compared to LPS, ^e^*p* < 0.005 compared to LPS, ^f^*p* < 0.001 compared to LPS, ^g^*p* < 0.0005 compared to LPS (adjusted *p*-values).

An opposing pattern of stimulation is obtained when studying PPARα mRNA expression (Figures 5A–H), whose translation product is involved in the breakdown of fatty acids. LPS-induced stimulation of PPARα mRNA was very low, contrasting with the robust increase of Srepb-1c mRNA. High PPARα mRNA was achieved in conditions of preconditioning with Vitamin D_3_ for 1 day prior to LPS stimulation of J774, whereas this required 5 days preincubation for HepG2 (Figures 5A,B). There was a notable delay in PPARα mRNA response in HepG2 cells preconditioned with Vitamin D_3_ (Figure 5B). A dose dependent response in PPARα mRNA expression is recorded when stimulating J774 or HepG2 with LPS and subsequently with Vitamin D_3_ or DHA. The highest levels of PPARα mRNA were achieved at 5 days' preincubation of J774 or HepG2 with 32 μg/ml DHA (Figures 5C,D). After LPS stimulation, Vitamin D_3_ (4 μg/ml) was able to increase PPARα mRNA expression significantly after 5 day's treatment in J774 and HepG2 cells (Figures 5E,F). One day's *post-hoc* stimulation with DHA (after LPS stimulation) at doses of 16 and 32 μg/ml was more efficient at increasing PPARα mRNA in J774 than in HepG2 (Figures 5G,H).

**Figure 6:**
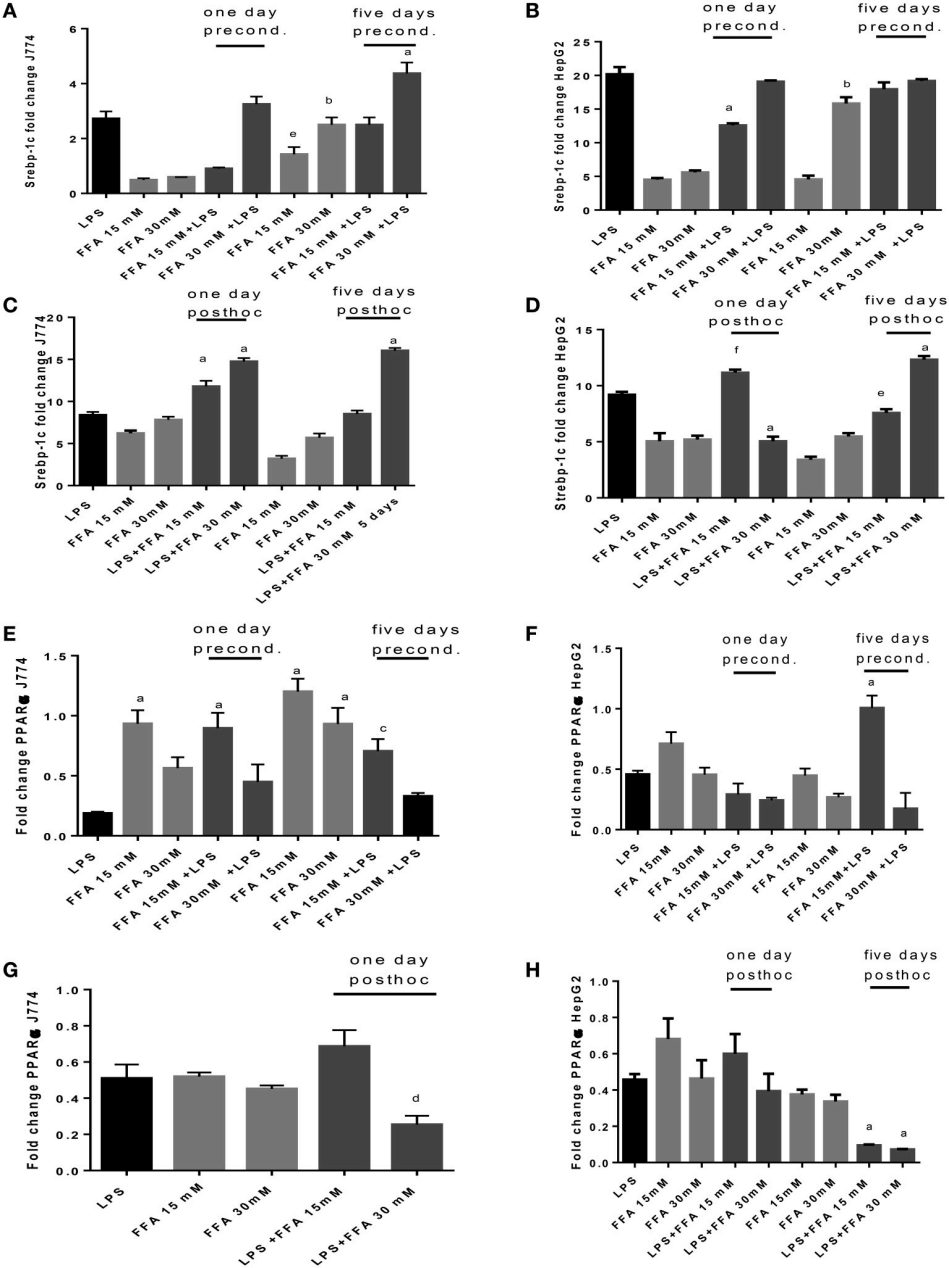
**(A–H)** qPCR analysis of J774 **(A,C,E,G)** and HepG2 **(B,D,F,H)** for Srebp-1c **(A–D)** and PPARα **(E–H)** mRNA expression induced by LPS and modulated by preconditioning with FFA **(A–D)** or *post-hoc* (subsequent to LPS) stimulation with FFA **(E–H)**. Triplicate data were averaged, normalized to GAPDH mRNA abundance and expressed in relation to baseline Srebp-1c or PPARα mRNA expression. ^a^*p* < 0.0001 compared to LPS, ^b^*p* < 0.0001 compared to day 1, ^c^*p* < 0.001 compared to LPS, ^d^*p* < 0.005 compared to LPS, ^e^*p* < 0.005 compared to day 1, ^f^*p* < 0.002 compared to LPS (adjusted *p*-values).

The sole addition of FFA without LPS led to significantly increased levels of Srebp-1c mRNA expression at 5 days' stimulation compared to 1 day stimulation of J774 cells. FFA at 30 mM increased Srepb-1c mRNA compared to LPS-induced expression significantly when added for 5 days to J774 culture prior to LPS stimulation (Figure 6A). While FFA at 30 mM significantly increased Srebp-1c mRNA at 5 days' stimulation compared to the shorter incubation period of 1 day, additional stimulation with LPS did not further increase Srebp-1c mRNA in HepG2 cells (Figure 6B). Prior stimulation with LPS of J774 and addition of FFA for 1 and 5 days significantly increased Srebp-1c mRNA (Figure 6C). HepG2 cells showed an inconsistent pattern of changes in Srebp-1c mRNA in response to LPS and subsequent stimulation with FFA at 15 and 30 mM (Figure 6D). FFA given for 1 and 5 days had a significant role in increasing PPARα mRNA levels. Subsequent LPS stimulation decreased this effect (Figure 6E). HepG2 cells showed an inconsistent pattern of changes in PPARα mRNA in response to FFA and subsequent stimulation with LPS (Figure 6F). Subsequent stimulation of J774 with FFA after 4 h LPS led to modulation of PPARα mRNA (Figure 6G); a clear reduction in PPARα mRNA was observed in HepG2 cells exposed to 15 mM FFA after LPS stimulation (Figure 6H).

## Discussion

Endotoxemia is an important factor in the progression from uncomplicated NAFLD to NASH (Finelli and Tarantino, 2014), which carries the risk of developing cirrhosis. This study analyses in parallel two TLR4 positive cell types of relevance to the liver, the macrophage and hepatocyte, for their regulation of inflammatory, fibrotic, and steatotic reactants when stimulated with Vitamin D_3_ or DHA. These are dietary immune modulators (Simopoulos, 2002; Prietl et al., 2013) whose adjuvant clinical utility is beginning to be widely studied in preclinical and clinical studies. The interest of this study lay in investigating a qualitative difference in ameliorating the effect of endotoxin if Vitamin D_3_ or DHA were present prior to this stimulus or available shortly after. This might give an indication whether constant dietary admixture is recommendable or whether doses of treatment after LPS stimulus were effective. TNFα is a key factor in the development of NAFLD and NASH in both humans and animals (Braunersreuther et al., 2012). Hasegawa et al. showed that TGFβ1 levels were increased in patients with NASH as compared to patients with hepatic steatosis (Hasegawa et al., 2001). Macrophages determine progression of inflammation. This is because activation of macrophages in liver leads to production of inflammatory mediators such as TNF, Interleukin-1 and reactive oxygen species in NASH. These inflammatory mediators further stimulate hepatocytes and hepatic satellite cells to induce hepatocyte steatosis and fibrosis. Macrophages encourage development of steatohepatitis because of the interaction of chemokine-chemokine receptors on liver cells and Kupffer cells (Miura et al., 2012). Steatosis, liver injury and proinflammatory monocyte infiltration was reduced *in vivo* as a result of depletion of Kupffer cells (Tosello-Trampont et al., 2012).

This study finds that preconditioning with Vitamin D_3_ or DHA prior to LPS stimulation or incubation following LPS stimulation (“*post-hoc*”) decreased TNFα and TGFβ production of J774 and of HepG2 significantly. Vitamin D_3_ and DHA signal via distinct receptors (VDR and GPR120) but affect TLR4 mediated signaling effects in a similar manner, whether allowing cells to adapt to the presence of Vitamin D_3_ or DHA prior to LPS stimulation or treating the inflammatory state of HepG2 or J774 induced by LPS with Vitamin D_3_ or DHA subsequently.

Stimulation with obesity relevant FFA elicits elevated levels of inflammatory cytokines in HepG2 and J774. The use of FFA allowed consideration of development of tolerance in our system; because FFA bind to TLR4 as endogenous DAMP, it is possible that there is dampening of LPS mediated effects on subsequent challenge or, vice versa, dampening of FFA effect because of prior TLR4 engagement with LPS. In our study, there might be evidence of tolerance development when stimulating J774 with LPS, then subsequently with FFA for 1 day, because the elicited TNFα response compared to that of FFA stimulation alone and was half that achieved by LPS stimulation alone (Figure 3C). This was not observed for HepG2.

Vitamin D_3_ and DHA efficiently downregulated LPS-induced Srebp-1c mRNA expression in J774 and HepG2 cells regardless of the timing of their addition, whether Vitamin D_3_ and DHA were present before (preconditioning) or after (*post-hoc*) LPS stimulation. The translation product of Srebp-1c mRNA is involved in increasing cholesterol biosynthesis as part of an acute phase reaction (Diomede et al., 2000). PPARα mRNA expression showed an inverse pattern to that observed for Srebp-1c mRNA, with regard to LPS-induced regulation and modulation by Vitamin D_3_ and DHA. Our data suggest that qPCR can detect a high ratio of Srebp-1c/PPARα in LPS-stimulated HepG2 and J774 which is viewed as unfavorable in the obese (Pettinelli et al., 2009) and is provoked by the addition of FFA in our study. Hepatic Srebp-1c mRNA levels were significantly elevated, while hepatic PPARα mRNA levels were significantly lower in obese patients undergoing bariatric surgery compared to non-obese patients undergoing cholecystectomy (Pettinelli et al., 2009). Relevant to our study, these obese patients with NAFLD had significantly lower DHA in liver total lipids compared to their study controls (Pettinelli et al., 2009).

Taken together, Vitamin D_3_ and DHA exerted beneficial effects in the context of endotoxin stimulation by reducing TNFα and TGFβ proteins, and Srebp-1c mRNA, whilst increasing PPARα mRNA. The translation products of Srebp-1c and PPARα mRNA (which were not measured) are involved in cholesterol biosynthesis and triglyceride turnover, respectively. Macrophage responses are skewed toward a phenotype that produces less proinflammatory TNFα and profibrotic TGFβ, thereby is less able to support NAFLD development and progression to NASH (Dey et al., 2014). Both, lipid metabolism and inflammation are relevant in the development of insulin resistance (Glass and Olefsky, 2012). Our data lend support to both, curative and preventative dietary regimes involving Vitamin D_3_ or DHA to quench the detrimental effects of low grade endotoxemia. *In vivo* studies are needed next to address the efficacy of dietary supplementation with an immune modulator in enhancing the outcome from NAFLD. One recent clinical trial of supplementation with Vitamin D_3_ showed significant improvement in some serum markers of inflammation, but a greater observation time (>4 months) was likely to be necessary to observe amelioration in transaminase levels and ultrasonographic grades of NAFLD (Sharifi et al., 2014). A meta-analysis of treatments of patients with NAFLD (for a median duration of 6 months) with supplements of omega 3 fatty acids showed significant ultrasonographic improvement of steatosis, likely involving a reversal of the Srebp-1c/PPARα ratio (Parker et al., 2012).

## Author contributions

RK, Substantial contributions to the acquisition, analysis, and interpretation of data for the work, drafting the work, final approval of the version to be published, agreement to be accountable for all aspects of the work in ensuring that questions related to the accuracy or integrity of any part of the work are appropriately investigated and resolved. JH, Substantial contributions to the conception or design of the work, final approval of the version to be published, agreement to be accountable for all aspects of the work in ensuring that questions related to the accuracy or integrity of any part of the work are appropriately investigated and resolved. CS, Substantial contributions to the interpretation of data for the work, drafting the work and revising it critically for important intellectual content, final approval of the version to be published, agreement to be accountable for all aspects of the work in ensuring that questions related to the accuracy or integrity of any part of the work are appropriately investigated and resolved.

## Funding

This study was funded by HCDP (Human Capacity Development Program) Kurdistan region government (award to RK).

### Conflict of interest statement

The authors declare that the research was conducted in the absence of any commercial or financial relationships that could be construed as a potential conflict of interest.
