# Supplementary material for: In vitro Modulation of the LPS-Induced Proinflammatory Profile of Hepatocytes and Macrophages- Approaches for Intervention in Obesity?
Source: Front Cell Dev Biol. 2016 Jun 22;4:61. doi: 10.3389/fcell.2016.00061 (PMC4916220; doi:10.3389/fcell.2016.00061)
Supplement: Supplementary file 1 [file Table1.docx]

Supplementary table 1. Sequences of primer pairs (with their annealing temperatures) used in this study

| Target | Forward sequences 5'-3' | Reverse sequences 5'-3' | Annealing  Temp.  ^0^C | Species |
| --- | --- | --- | --- | --- |
| GAPDH | CCTGGAGAAACCTGCCAAGTATG | AGAGTGGGAGTTGCTGTTGAAGTC | 55 | Mouse |
| TNF-α | GGCAGGTCTACTTTGGAGTCATTCC | ACATTCGAGGCTCCAGTGAATTCGG | 60 | Mouse |
| TLR4 | CGCTTTCACCTCTGCCTTCACTACAG | ACACTACCACAATAACCTTCCGGCTC | 55 | Mouse |
| SREBP-1C | TCTGCCTTGATGAAGTGTGG | AGCAGCCCCTAGAACAAACA | 55 | Mouse |
| Vitamin D R | TACATCCGCTGCCGCCACCCGC | TCAGGAGTCTCATTGCC | 55 | Mouse |
| PPAR-α | TCGAGGAAGGCACTACACC | TCTTCCCAAAGCTCCTTCAA | 55 | Mouse |
| Insulin R | TTTGTCATGGATGGAGGCTA | CCTCATCTTGGGGTTGAACT | 55 | Mouse |
| TNF-α | GGACCTCTCTCTAATCAGCCCTC | TCGAGAAGATGATCTGACTGCC | 55 | Human |
| PPAR-α | GCAGAAACCCAGAACTCAGC | ATGGCCCAGTGTAAGAAACG | 55 | Human |
| SREBP-1C | GGATTGCACTTTCGAAGACATG | ACTCTGGACCTGGGTGTGCAAG | 55 | Human |
| TLR4 | AGGATGATGCCAGGATGATGTC | TCAGGTCCAGGTTCTTGGTTGAG | 55 | Human |
| GAPDH | TCCCTGAGCTGAACGGGAAG | GGAGGAGTGGGTGTCGCTGT | 55 | Human |
| Vitamin D R | CTCATCTGTCAGAATGAACTCCTTCA | TCACCAAGGACAACCGACG | 55 | Human |
| Insulin R | AACCAGAGTGAGTATGAGGAT | CCGTTCCAGAGCGAAGTGCTT | 60 | Human |
