# Supplementary material for: In vitro Modulation of the LPS-Induced Proinflammatory Profile of Hepatocytes and Macrophages- Approaches for Intervention in Obesity?
Source: Front Cell Dev Biol. 2016 Jun 22;4:61. doi: 10.3389/fcell.2016.00061 (PMC4916220; doi:10.3389/fcell.2016.00061)
Supplement: Supplementary file 2 [file Image1.pdf]

# Supplementary

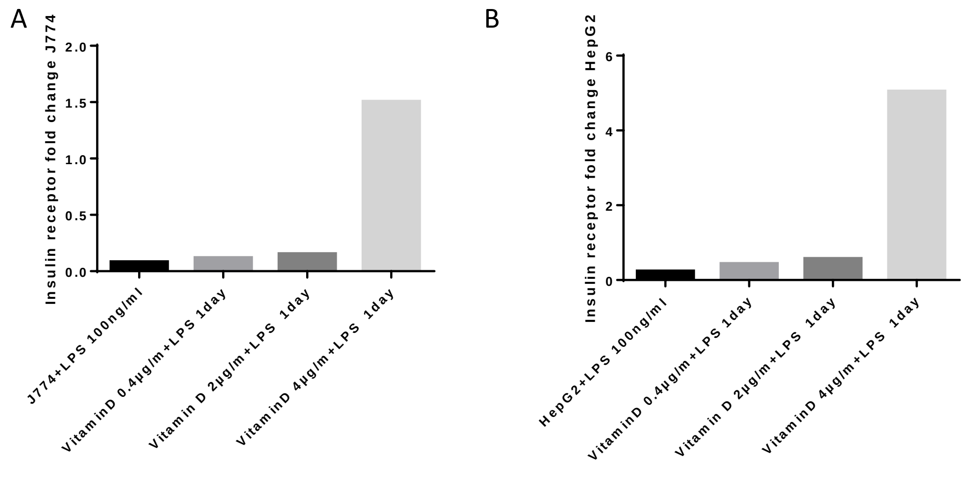


Supplementary figure 1: Cells (A, J774; B, HepG2) were preconditioned with Vitamin D for one day, then stimulated with LPS for 24 hrs, RNA was prepared using TriReagent and qPCR for insulin receptor gene performed on cDNA transcribed from 3µg RNA each.

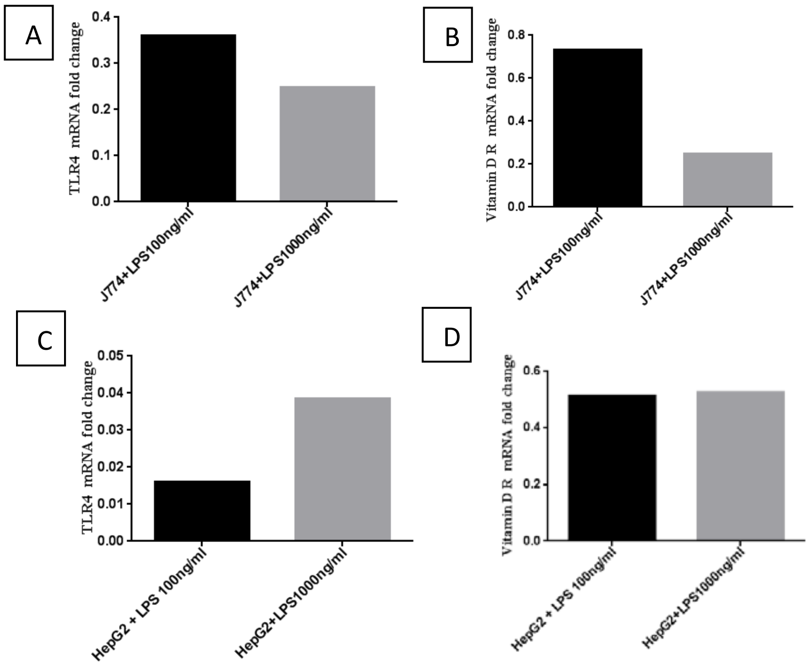


Supplementary figure 2
Cells (J774, A, B; HepG2, C, D) were stimulated for 24 hrs, RNA was prepared using TriReagent and qPCR for TLR4 (A, C) and VDR (B, D) mRNA expression performed on cDNA transcribed from 3 μg RNA each.

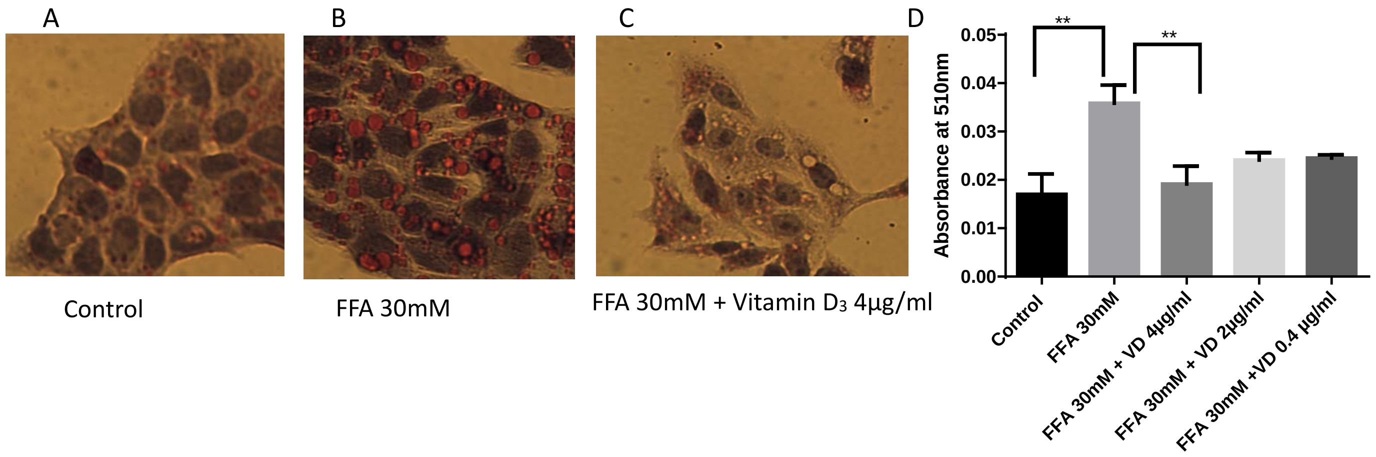


Supplementary 3: Micrographs (x40 oil) of Oil Red O stained HepG2 cells grown on coverslips and exposed to FFA or FFA with Vitamin $D_3$ for 48 hours (A-C). Spectrophotometric analysis of DMSO solubilised cells after washing; mean ± SD of 2 independent experiments is presented (unpaired t-test, $p<0.05$).
